# The *Arabidopsis* chromatin regulator MOM1 is a negative component of the defense priming induced by AZA, BABA and PIP

**DOI:** 10.3389/fpls.2023.1133327

**Published:** 2023-05-09

**Authors:** Julián O. Miranda de la Torre, Micaela Y. Peppino Margutti, Ignacio Lescano López, Damián Alejandro Cambiagno, María E. Alvarez, Nicolás M. Cecchini

**Affiliations:** ^1^ Centro de Investigaciones en Química Biológica de Córdoba, CIQUIBIC, Consejo Nacional de Investigaciones Científicas y Técnicas (CONICET), Departamento de Química Biológica-Ranwel Caputto, Facultad de Ciencias Químicas, Universidad Nacional de Córdoba, Córdoba, Argentina; ^2^ Unidad de Estudios Agropecuarios (UDEA), Instituto Nacional de Tecnología Agropecuaria (INTA)- Consejo Nacional de Investigaciones Científicas y Técnicas (CONICET), Córdoba, Argentina; ^3^ Departamento de Química Biológica-Ranwel Caputto, Facultad de Ciencias Químicas, Universidad Nacional de Córdoba, Córdoba, Argentina

**Keywords:** *Arabidopsis*, azelaic acid, pipecolic acid, β-Aminobutyric acid, MOM1, plant defense, priming, chromatin

## Abstract

In plants, the establishment of broad and long-lasting immunity is based on programs that control systemic resistance and immunological memory or “priming”. Despite not showing activated defenses, a primed plant induces a more efficient response to recurrent infections. Priming might involve chromatin modifications that allow a faster/stronger activation of defense genes. The *Arabidopsis* chromatin regulator “*Morpheus Molecule 1*” (MOM1) has been recently suggested as a priming factor affecting the expression of immune receptor genes. Here, we show that *mom1* mutants exacerbate the root growth inhibition response triggered by the key defense priming inducers azelaic acid (AZA), β-aminobutyric acid (BABA) and pipecolic acid (PIP). Conversely, *mom1* mutants complemented with a minimal version of MOM1 (*miniMOM1* plants) are insensitive. Moreover, *miniMOM1* is unable to induce systemic resistance against *Pseudomonas* sp. in response to these inducers. Importantly, AZA, BABA and PIP treatments reduce the *MOM1* expression, but not *miniMOM1* transcript levels, in systemic tissues. Consistently, several MOM1-regulated immune receptor genes are upregulated during the activation of systemic resistance in WT plants, while this effect is not observed in *miniMOM1*. Taken together, our results position MOM1 as a chromatin factor that negatively regulates the defense priming induced by AZA, BABA and PIP.

## Introduction

To survive pathogens and pests attack, plants depend on physical barriers and an efficient innate immune system based on the ability to sense foreign or self-modified molecules (Spoel and Dong, 2012; Rhodes et al., 2022). Recognition of pathogens mostly relies on two types of proteins. The pattern recognition receptors (PRRs) are receptor-like kinases/proteins that reside in the plasma membrane and perceive apoplastic molecular patterns from microbes (MAMPs/PAMPs; microbe-/pathogen-associated molecular patterns-) (Macho and Zipfel, 2014). In addition, the nucleotide-binding leucine-rich repeat receptors (NLRs), are proteins that recognize specific pathogen effectors at the intracellular level (Jones et al., 2016). The activation of PRR and NLR receptors not only triggers local defenses but can also induce a systemic and broad-spectrum non-autonomous immunity, usually associated with a state of alert or immunological memory defined as “priming*”* (Parker, 2009; Fu and Dong, 2013; Pieterse et al., 2014; Conrath et al., 2015).

A primed plant does not generally exhibit induced/activated defenses, but instead responds faster, stronger and/or in a more sustained manner to a second infection or new challenge. This primed state induction is of low energy cost and, thus, it is believed to increase the fitness of the plants growing under biotic stress conditions (Martinez-Medina et al., 2016; Mauch-Mani et al., 2017). The systemic resistance and the associated priming might be triggered by a first local stimulus in leaves or roots inducing different types of induced systemic resistance (ISR) programs (Pieterse et al., 2014; De Kesel et al., 2021). Among them, one of the best characterized is the systemic acquired resistance (SAR) activated by a necrotizing pathogen infection (Fu and Dong, 2013; Conrath et al., 2015). Furthermore, exogenous treatment with some plant-produced defense compounds like azelaic acid (AZA), β-aminobutyric acid (BABA), and pipecolic acid (PIP) or its active derivative N-hydroxy-PIP (NHP), are capable of inducing a primed state without activating direct defense responses (e.g. *PR1* defense gene induction) (Zimmerli et al., 2000; Ton et al., 2005; Jung et al., 2009; Návarová et al., 2012; Chen et al., 2018; Hartmann et al., 2018; Wang et al., 2018; Cecchini et al., 2019). Some of these molecules are also proposed as systemic signals required for SAR and/or ISR induction [e.g., AZA and PIP/NHP; (Vlot et al., 2021)].

The establishment and maintenance of the primed state may involve chromatin alterations, accumulation of inactive signaling kinases (e.g. MAPKs), and changes in the amount and/or location of immune receptors for a more efficient defense response (e.g. FLS2 and CERK1) (Beckers et al., 2009; Tateda et al., 2014; Cecchini et al., 2015a; Conrath et al., 2015; Tsuda and Somssich, 2015; Baum et al., 2019). Many studies have focused on chromatin alterations associated to transcriptional activation of defense genes due to epigenetic changes affecting marks of 5-methylcytosine (5mC) in DNA, histone modifications (Jaskiewicz et al., 2011; López et al., 2011; Luna et al., 2012; Singh P. et al., 2014; Conrath et al., 2015; Martinez-Medina et al., 2016), or production of small RNAs (sRNA) for (post) transcriptional gene regulation (Wilkinson et al., 2019). Thus, it is probable that diverse enzymes or epigenetic components together with chromatin remodeler complexes play key roles in the induction of the primed state(s). However, there is scarce evidence of the molecular basis or factors being directly implicated in the priming of the plant immune system (López et al., 2011; Mozgová et al., 2015; Crisp et al., 2016; Lämke and Bäurle, 2017; Alonso et al., 2019; Wilkinson et al., 2019; Cambiagno et al., 2021; Hannan Parker et al., 2022).

Recently, it was suggested that the *Arabidopsis* chromatin and transcriptional gene silencing (TGS) regulator “*Morpheus Molecule 1*” (MOM1) maintains repression of defense priming (Cambiagno et al., 2018; Cambiagno et al., 2021). *mom1* mutants have enhanced resistance to *Pseudomonas syringae* pv. *tomato* (*Pst*), without constitutive expression but with a predisposition to activate the defense genes PATHOGENESIS RELATED GENE 1 (*PR1*) and ISOCHORIMATE SYNTHASE 1 (*ICS1/SID2*) (Cambiagno et al., 2018), phenocopying a primed state (Cambiagno et al., 2021). MOM1 is a plant-specific protein carrying part of the SNF2 domain present in many ATP-dependent chromatin remodelers. MOM1 represses a subset of transposon elements (*TE*s), mostly pericentromeric *TE*s (p*TE*s), which are also targeted by RNA-directed DNA methylation (RdDM). This effect is independent on DNA methylation as *mom1* mutant release repression of the p*TE TSI* without changing its 5mC level (Amedeo et al., 2000; Habu et al., 2006; Vaillant et al., 2006; Numa et al., 2010; Yokthongwattana et al., 2010; Nishimura et al., 2012; Cambiagno et al., 2018). It is currently unknown how MOM1 mediates transcriptional silencing, but this may involve it as an adapter of a multi-protein complex repressing heterochromatin, rather than acting as an ATP-dependent chromatin remodeler (Han et al., 2016).

The increased resistance observed in *mom1* plants is proposed to be the consequence of a co-regulation of the p*TEs* and unlinked *PRR* and *NLR* genes via “common” sRNAs with perfect match to both loci (Cambiagno et al., 2018). During development, *mom1* mutants increase the expression of numerous *NLR*/*PRR* genes (the so-called *MOM1-NLR/PRRs*), including the well-characterized RECEPTOR-LIKE KINASE7 (*RLK7*) and ACTIVATED DISEASE RESISTANCE 1 (*ADR1*) proteins (Habu et al., 2006; Bonardi et al., 2011; Hou et al., 2014; Cambiagno et al., 2018; Hou et al., 2019; Jubic et al., 2019). The expression of truncated versions of MOM1 in the *mom1* background indicated that the minimal version of MOM1 that is sufficient to reset *TSI* silencing is a polypeptide carrying the nuclear localization signal (NLS) and the C-terminal domain of 197 amino acids of MOM1 (NLS + ‘conserved MOM1 motif 2’; CMM2; *miniMOM1* plants) (Čaikovski et al., 2008; Mlotshwa et al., 2010; Nishimura et al., 2012; Cambiagno et al., 2018). Moreover, *miniMOM1* plants also restore the susceptibility to *Pst* and the repression of *NLR/PRRs* and *PR1* genes, suggesting that silencing of *TE*s by MOM1 affects biotic defenses (Cambiagno et al., 2018).

Here, we investigated if MOM1 plays a role in defense priming and systemic resistance induction. For this, we made use of the well-known priming inducers AZA, BABA, and PIP in *in vitro* and *in planta* assays. We show that the *mom1* mutant display stronger responses to all three inducers while the *miniMOM1* plants are insensitive to them. Importantly, wild-type plants treated with these priming inducers, reduce the *MOM1* gene expression in systemic tissues. In addition, many of the MOM1*-*dependent *NLR/PRRs* are induced in PIP- and SAR-primed plants. Based on these results we propose that MOM1 functions as a negative regulator of defense priming against pathogens triggered by AZA, BABA, and PIP in *Arabidopsis*.

## Materials and methods

### Plants and growth conditions

*Arabidopsis thaliana* ecotypes Columbia-0 (Col-0) and Zürich (Zu), and the mutant line *fmo1-1* (SALK_026163) were obtained from the Arabidopsis Biological Resource Centre (Ohio State University, Columbus, OH, USA)*. mom1-1* mutant and *miniMOM1* transgenic plants were provided by Dr. Jerzy Paszkowski (The Sainsbury Laboratory) and Dr. Ortrun Mittelsten Scheid (Gregor Mendel Institute) (Nishimura et al., 2012; Moissiard et al., 2014).

For the studies with seedlings, sterile seeds were imbibed and stratified for 4 days at 4°C, germinated, and grown in ½ Murashige–Skoog (MS) 0.8% agar plates with 1% sucrose in a growth chamber with 12 h light (100–120 µmol sec^-1^ m^-2^) and 12 h dark at 20–22°C (Cecchini et al., 2022). For optimal controlled growing conditions and gas exchange, the plates were wrapped with paper tape as described by Xu et al. (2019). The four-week-old adult plants were germinated and grown on soil under 12 h light (100–120 µmol sec^-1^ m^-2^) and 12 h dark cycles at 20-22°C.

### Treatment of seedlings with priming inducers in plate assays

Seedlings were exposed to the priming inducers as previously described with some modifications (Wu et al., 2010; Cecchini et al., 2019). Briefly, seeds were germinated and grown vertically on ½ MS (1% sucrose) agar plates supplemented with 20 and 40 µM of azelaic acid (AZA; C_9_H_16_O_4_, Sigma-Aldrich), 25 and 75 µM of 3-aminobutanoic acid (BABA; C_4_H_9_NO_2_, Sigma-Aldrich), 0.75 and 1.25 mM of pipecolic acid (PIP; C_6_H_11_NO_2_, Sigma-Aldrich), or with water (mock). After 14 days the plates were scanned using a Hewlett-Packard Company *Photosmart 4070* scanner. Root lengths were analyzed using ImageJ/Fiji (Schindelin et al., 2012). The parameter ‘response gain effect’ was calculated to quantitatively evaluate the root inhibition variation due to inducer- with respect to mock- treatments as previously described (Jiang et al., 2021).

### Treatment of roots with priming inducers for systemic resistance assays

For systemic resistance assays, four-week-old *Arabidopsis* plants grown in trays were soil-drenched by immersing the pot for 30 minutes in 1mM AZA, 300 µM BABA, 1mM PIP solutions or water (mock), avoiding contact with aerial tissues (Cecchini et al., 2019). Trays were then allowed to drain off the excess of solutions for 1-2 minutes before being returned to the growth chamber. One day later, plants were syringe-inoculated in leaves with a virulent *Pseudomonas cannabina* pv. *alisalensis* [*Pma*DG3; formerly called *P. syringae* pv. *maculicula* (Guttman and Greenberg, 2001; Bull et al., 2010; Baltrus et al., 2011)] (OD_600 = _0.005). Bacterial growth quantification was done by dilution-plating using at least 5 leaves from 5 different plants 3 days after bacteria inoculation. The parameter ‘response gain effect’ was calculated to quantitatively evaluate the resistance variation due to inducer- with respect to mock- treatments as previously described (Jiang et al., 2021).

### Gene expression analysis

To analyze the transcript levels on plants growing on plates, samples (roots and aerial part from at least 3 seedlings) were collected after 25 or 35 days and immediately frozen. For systemic resistance assays three leaves from three different plants were collected and pooled together per independent experiment one day after root application of inducers and immediately frozen.

Total RNA was extracted using SDS-LiCl RNA purification protocol (Verwoerd et al., 1989). Total RNA (1 µg) was treated with RQ1 DNAase (Cat. #M6101, Promega) and then incubated with random hexamer and oligo(dT) primers (9:1 ratio) and M-MLV retro-transcriptase (Cat. #M1701, Promega) to synthesize cDNA according to manufacturers’ procedures, as previously described (Cambiagno et al., 2018; Cecchini et al., 2022). Transcript levels were analyzed by reverse transcription followed by quantitative PCR (RT-qPCR). We used 1:4 cDNA dilutions in 15 μL reactions of Luna Universal Dye qPCR Master Mix (New England Biolabs) on a CFX96 Touch™ real-time PCR System (Bio-Rad) and the following set up: 95°C for 1 min and 45 cycles at 95°C for 15 s and 60°C for 30 s, and 1 cycle of dissociation from 65°C to 95°C with 1°C temperature increase for 5s. Sequences of the oligonucleotide used as primers are shown in **Supplementary Table 1**. The raw data obtained with CFX Manager 3.1 Software (Bio-Rad) was baseline corrected, and the window of linearity was determined using LinRegPCR 2021.1 (Ruijter et al., 2009). EF1α (elongation factor 1 alpha, At5g60390) was used as a reference gene.

### mRNA-Seq analysis

mRNA-seq datasets from systemic leaves of root-treated plants with PIP or NHP (Hartmann et al., 2018; Yildiz et al., 2021) or leaf-infiltrated with *Pseudomonas syringae* pv. *maculicola* to induce SAR (Bernsdorff et al., 2016; Baum et al., 2019) were analyzed using the raw data available on the following BioProyects: PRJEB23627, PRJEB43717, PRJEB32929, and PRJEB12204. Trimmed reads were mapped to the TAIR10 genome (*Arabidopsis thaliana*) with HISAT2 (Kim et al., 2015) and counts were generated with featureCounts (version 1.6.2) (Liao et al., 2014). Differential expression of *PRR/NLR* genes (Cambiagno et al., 2018; Cambiagno et al., 2021) were determined with Deseq2 R package (version 1.20.0) (Love et al., 2014) considering a False Discovery Rate (FDR) lower than 0.05 and log_2_ Fold Change (FC) higher than 1. Commonly up-regulated *PRR/NLR* genes between samples (intersections) were shown by using the UpSet plots R package (Conway et al., 2017). Counts of *PRR/NLR* genes activated in each sample were plotted using ggplot2 (Wickham, 2016; https://ggplot2.tidyverse.org).

### Statistical analysis

Analyses were done using SigmaPlot v11.0 (Systat Software, Inc.) and InfoStat statistical software v2018 (www.infostat.com.ar; Grupo InfoStat). Outliers were excluded using Grub’s test (α = 0.05). The normality of the data was tested by Shapiro Wilk’s test. Analysis of variance (ANOVA) or Kruskal-Wallis nonparametric analysis followed by *post hoc* tests were used for significant differences as indicated in Figure legends. Square root-transformed data was used for root length and bacterial growth curves in normality and ANOVA tests.

## Results

### *mom1* plants show no defense activation under optimal and germ-free growth conditions

**Figure 1:**
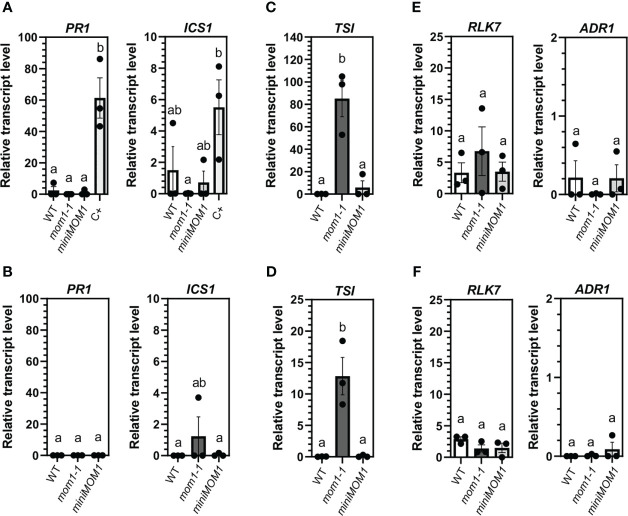
Defense gene activation in WT (Zu), *mom1-1*, and *miniMOM1* plants under optimal and sterile growth conditions. Seeds were germinated and grown *in vitro* as described in Materials and Methods. After 25 **(A, C, E)** or 35 days **(B, D, F)** post-germination the relative transcript levels of *PR1* and *ICS1* **(A, B)**, *TSI* **(C, D)** and *RLK7* and *ADR1* **(E, F)** genes were analyzed by RT-qPCR. In **(A)** *Pma*DG3 infected wild-type plants were used as positive control (C+). Values represent the average +/- standard error of three independent experiments (each data point with at least 3 different seedlings pooled together for RNA extraction). *ELF1α* was used as a reference gene. Individual data points are presented as scatter-dots. Different letters indicate significant differences among samples (P < 0.05, ANOVA, Tukey’s multiple comparison test).

Uninfected *mom1* mutant plants induce several *NLR/PRRs* genes (*MOM1-NLR/PRRs*) and the defense marker genes *ICS1* and *PR1* as they develop (Cambiagno et al., 2018). The cause of such induction is unknown but was suggested to be associated to hypersensitivity to stresses and/or to aging since at young stages *mom1* plants expressed *TSI* but not defense genes (Steimer et al., 2000; Cambiagno et al., 2018). To better analyze this, *mom1-1* plants were grown under optimal *in vitro* conditions of gas exchange and minimal stress by using air-permeable paper-tape to seal the plates as previously reported (Xu et al., 2019). We then quantified defense gene transcripts at 25 and 35 days post-germination, to evaluate plant responses until the flowering transition stage. The *miniMOM1* plants were also examined as they complement the *mom1-1* induction of defense genes (Cambiagno et al., 2018). As observed in **Figures 1A, B**, we found no differences on the levels of *PR1* and *ICS1* expression in either *mom1-1* or *miniMOM1* compared to wild-type plants. The infected wild-type plants used as a positive control (C+) showed a strong induction of both genes. We also tested the expression of the p*TE TSI* in these samples and detected its induction in *mom1-1*, but not in *miniMOM1*, as expected (**Figures 1C, D**). Then, we examined the expression of the *MOM1-NLR/PRRs* genes *RLK7* and *ADR1* (Cambiagno et al., 2018). We found no changes in *ADR1* transcript levels relative to wild-type plants, and a slight but no significant increase of *RLK7* in 25 days-old plants (**Figures 1E, F**).

These results indicate that the *mom1* mutants do not activate the defense genes when growing under optimal conditions for at least 35 days, suggesting that undefined stress conditions, but not aging, lead to defense induction in this mutant.

### *mom1* and *miniMOM1* roots respond differently to AZA, BABA and PIP

**Figure 2:**
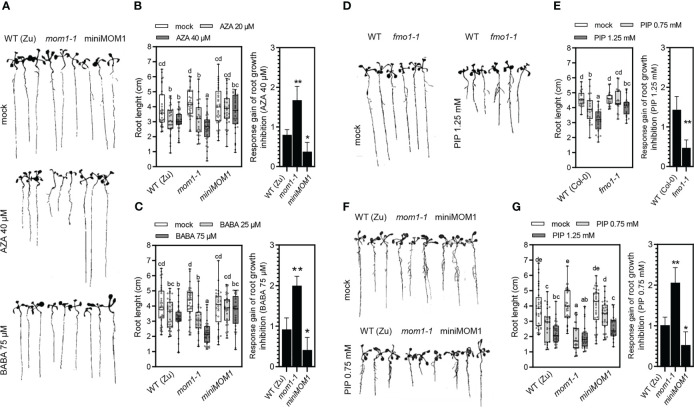
Effects of the priming inducers azelaic acid (AZA), β-aminobutyric acid (BABA) and pipecolic acid (PIP) on roots growth. **(A, D, F)** Representative images showing the effects of AZA, BABA and PIP or mock on the principal root length in different genotypes. **(B, C, E, G)** Quantification of the principal root length of 14-day-old WT (Zu, Col-0), *mom1-1*, miniMOM1 and *fmo1-1* seedlings grown on vertical agar media plates supplemented with different concentrations of AZA, BABA or PIP and mock. All data points (biological replicates) are presented as scatter-dots in the boxplots. Right graphs: average +/- standard error of the response gain of root growth inhibition (mock minus inducer treatments) calculated with the data obtained in **(B, C, E, G)** as previously described (Jiang et al., 2021). The data of 5 independent experiments (each with at least 5 **(B, C)** and 4 **(E)** biological replicates; n = 25-20). The different letters (P < 0.05, ANOVA, Fisher’s LSD test) and the asterisks (**p < 0.05 or *p < 0.1, Student’s *t* test) indicate statistically significant differences.

If MOM1 is a regulator of the primed state, *mom1* plants might respond differentially to priming inducers. In *Arabidopsis* the induction of defense priming by AZA and BABA is related with their inhibition effect of the primary root growth (Wu et al., 2010; Bouain et al., 2018; Cecchini et al., 2019). Therefore, we tested whether *mom1-1* roots respond distinctively to these inducers. To do this, wild-type, *mom1-1* and *miniMOM1* plants were germinated and grown in vertical plates with solid media supplemented with different concentrations of AZA (20 and 40 µM) and BABA (75 and 125 µM). Plants were grown under optimal conditions as described above, and after 14 days the principal root length was measured. As observed in **Figure 2A**, in both wild-type and *mom1-1*, root growth was inhibited in response to AZA and BABA compared to mock treatment. Root length quantification indicated that *mom1-1* is more susceptible to both inducers than wild-type plants (**Figures 2B, C**). This was particularly noticeable when considering the response gain analysis of the inducers inhibitory effect on roots (**Figures 2B, C** right graphs). Unexpectedly, we found no significant differences in *miniMOM1* root length in response to either inducer compared to mock treatments, even at the higher concentration supplied. Supporting this, the response gain revealed a reduced responsiveness in *miniMOM1* plants (**Figures 2B, C** right graphs).

Another important priming inducer is PIP (Návarová et al., 2012). However, its effect on root growth has not been tested so far. Thus, we first examined if different concentrations of PIP (0.75 and 1.25 mM) inhibit primary root growth in wild-type seedlings. In parallel, we tested the *fmo1-1* mutant plants that are impaired in the FLAVIN-DEPENDENT-MONOOXYGENASE1 (FMO1) enzyme involved in the conversion of PIP into the bioactive derivative NHP (Chen et al., 2018; Hartmann et al., 2018). As shown in **Figures 2D, E**, root growth was inhibited in wild-type plants by 1.25 mM of PIP. Interestingly, the *fmo1-1* mutants exhibited a reduced response under this condition (**Figures 2D, E**; and right graphs). This indicates that PIP is capable of inhibiting *Arabidopsis* root growth through a FMO1-dependent signaling pathway. Next, we analyzed the effect of PIP in *mom1-1* and *miniMOM1* and found that, similarly to the AZA and BABA treatments, *mom1-1* roots were more susceptible to the inducer compared to wild-type plants (**Figures 2E, F**; and right graphs). Contrary, in *miniMOM1* we only observed a diminished response to intermediate PIP concentration compared to the wild-type plants.

Taken together, these results show that root growth is normal in *mom1* mutants grown under optimal conditions but is markedly reduced in response to AZA, BABA and PIP compared to wild-type plants. This indicates that MOM1 maintains a negative regulation of priming inducers-mediated root growth inhibition. Furthermore, the results suggest that *miniMOM1* is a gain-of-function version of MOM1.

### Systemic resistance against *Pseudomonas* sp. induced by AZA, BABA and PIP is impaired in *miniMOM1* plants

**Figure 3:**
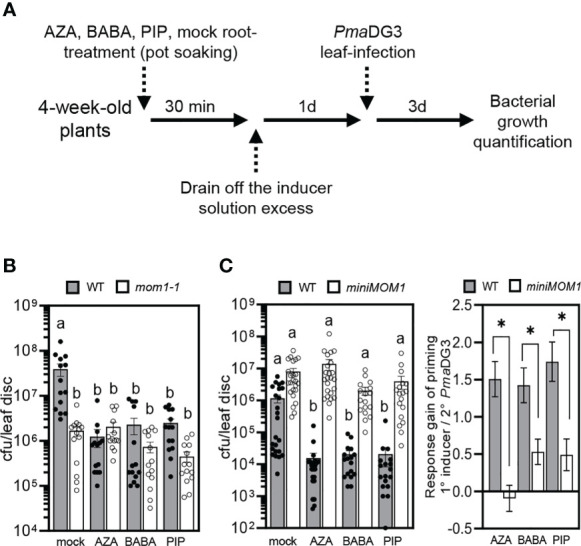
Induction of systemic resistance to bacterial infection by treatments with azelaic acid (AZA), β-aminobutyric acid (BABA) and pipecolic acid (PIP). **(A)** Priming treatment scheme. **(B, C)** Growth of the virulent bacteria *Pseudomonas cannabina* pv. *alisalensis* (*Pma*DG3) on WT (Zu), *mom1-1* **(B)** and *miniMOM1* **(C)** plants at 3 days post-infection. *Pma*DG3 was infiltrated in leaves 1 day after roots were soil-drenched with 1mM AZA, 300 µM BABA, 1mM PIP or mock solutions. Values represent average number of colony-forming units per leaf disc +/- standard error from three or four independent experiments (each one with at least 3 biological replicates; n = 14 in **(B)** and n = 20 in **(C)**). Individual data points (biological replicates) are presented as scatter-dots. Right graph in **(C)**: Response gain of the systemic priming associated with the root-applied AZA, BABA and PIP inducers (mock minus inducer treatments). The response gain was calculated with the data obtained in **(C)** as previously described (Jiang et al., 2021). Different letters (P < 0.05, ANOVA, Tukey’s multiple comparison test), and the asterisk (P < 0.05, Student’s *t* test) indicate statistically significant differences.

*mom1* mutants show a primed-like phenotype with enhanced resistance to *Pseudomonas* sp. and, thus, MOM1 is a proposed negative factor of the priming against pathogens (Cambiagno et al., 2018; Cambiagno et al., 2021). Then, if *miniMOM1* is a gain-of-function version of MOM1, the induction of systemic resistance by AZA, BABA and PIP might be affected in *miniMOM1* plants. To analyze this possibility, we evaluated the effect of these inducers on systemic protection against bacterial infection. We treated roots of adult wild-type, *mom1-1* and *miniMOM1* plants with 1 mM AZA, 300 µM BABA or 1mM PIP (or mock) solutions as indicated in **Figure 3A** and previously described (van Hulten et al., 2006; Návarová et al., 2012; Cecchini et al., 2019). After 1 day, we infected the leaves with a virulent strain of *Pseudomonas* sp. ([*Pma*DG3; (Guttman and Greenberg, 2001; Bull et al., 2010; Baltrus et al., 2011)], and quantified bacterial growth at 3 days post-infection. As expected, the treatment with AZA, BABA or PIP reduced the susceptibility of wild-type plants to *Pma*DG3 compared to mock treatment (**Figures 3B, C**). *mom1-1* was less susceptible to bacterial infection after mock treatment than WT plants (**Figure 3B**), confirming it has increased resistance as previously observed (Cambiagno et al., 2018; Cambiagno et al., 2021). Interestingly, the inducers did not improve resistance in the mutant, indicating that AZA, BABA or PIP are unable to enhance the mutant “basal” primed-like state. Contrary, *miniMOM1* plants showed no enhanced resistance to the bacteria in response to any of the inducers. Moreover, the response gain analysis for each treatment revealed a marked reduction of the priming level in *miniMOM1* compared to wild-type plants, particularly, for the AZA treatment (**Figure 3C**).

These results reinforce the notion that *miniMOM1* acts as a gain-of-function version of MOM1 during the induction of systemic resistance against pathogens mediated by AZA, BABA and PIP. In addition, they strongly support MOM1 as a negative regulator of priming and systemic resistance against *Pma*DG3.

### Treatments with AZA, BABA and PIP decrease *MOM1* transcripts in systemic leaves

**Figure 4:**
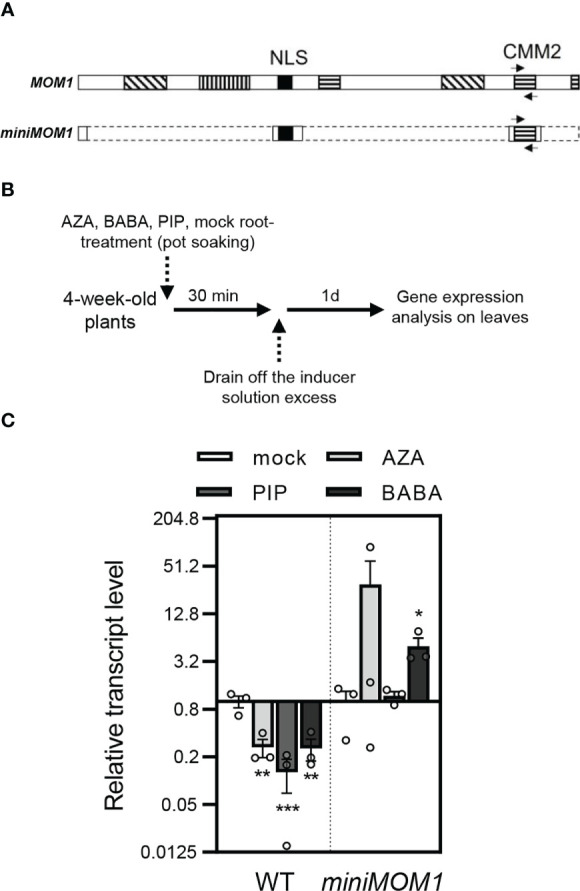
Expression of *MOM1* and *miniMOM1* (*CMM2* domain) in response to the priming inducers. **(A)** Schemes of the MOM1 and miniMOM1 proteins showing the CMM2 shared domain and nuclear localization sequence (NLS). Boxes with lined pattern indicate other conserved domains in MOM1. Arrows indicate the position of the primers designed for the transcriptional analysis the *CMM2.* The dashed lines in miniMOM1 indicate the deleted regions from MOM1. Adapted from Čaikovski et al. (2008). **(B)** Priming treatment schemes. **(C)** *CMM2* transcript levels quantified by RT-qPCR in WT (Zu) and *miniMOM1* leaves 1 day after root-applied soil-drenched with 1mM AZA, 300 µM BABA, 1mM PIP or mock solutions. Values represent average +/- standard error of three independent experiments (each data point with 3 leaves from 3 different plants pooled together for RNA extraction). *ELF1α* was used as a reference gene. Individual data points are presented as scatter-dots. Asterisks indicate significant differences with respect to mock (*p<0.1; **p<0.05; ***p<0.001, Kruskal-Wallis non-parametric analysis, Dunn’s test).

*MOM1* transcripts are slightly reduced during some bacterial infections and PAMP treatments [not shown; Genevestigator and eFP Browser (Winter et al., 2007; Hruz et al., 2008)]. Thus, we evaluated if priming inducing agents affected *MOM1* gene expression in wild-type plants. In parallel, we evaluated the expression of the *miniMOM1* transgene, which is controlled by the native *MOM1* promoter (Čaikovski et al., 2008). To do this, we used primers for RT-qPCR analysis of the *CMM2* domain, shared between the *MOM1* native transcript and the *miniMOM1* transgene (**Figure 4A**). Plants were soil-drenched with 1 mM AZA, 300 μM BABA or 1 mM PIP, and 24 hours later leaves were sampled to quantify gene expression as indicated in **Figure 4B**. As shown in **Figure 4C**, wild-type plants reduced the *MOM1* (*CMM2* domain) transcripts in response to all three inducers. In contrast, *miniMOM1* plants showed no significant changes in the *CMM2* transcript levels after AZA and PIP treatments (with a tendency to increase in response to AZA) but increased its content in response to BABA.

These results suggest that the downregulation of *MOM1* mRNAs is necessary to activate defense priming. This regulation is not merely controlled by the promoter present in the *miniMOM1* construct. Moreover, the finding that native *MOM1* and *miniMOM1* transcripts show different contents in response to the inducers, provides a putative explanation of the positive dominant effect observed in *miniMOM1* plants.

### MOM1-regulated immune receptors are induced in SAR and primed plants

**Figure 5:**
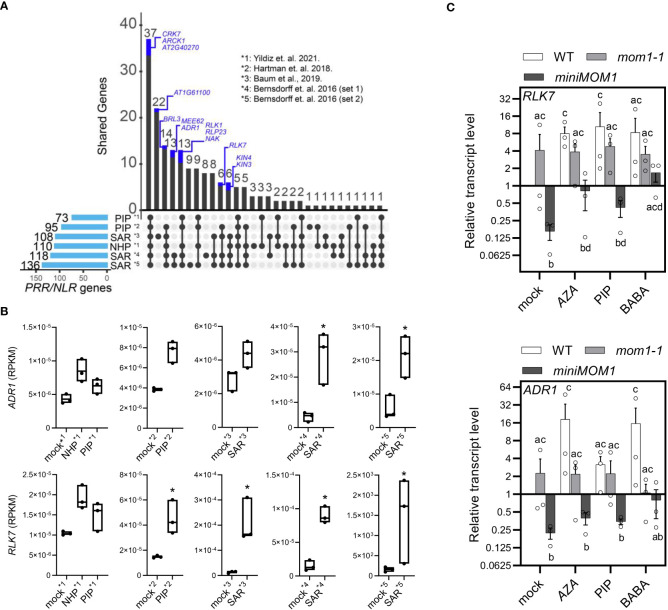
*MOM1-PRR/NLRs* induced in PIP/NHP- and SAR-primed plants. **(A)** UpSet plot showing the upregulated *PRR/NLR* shared genes (FDR < 0.05, FC > 1) between SAR-, PIP-, and NHP- treated samples. The number of activated genes in each dataset is described at the left and represented with light-blue bars. Intersections between samples are denoted by black lines linking black dots. The number of genes in the intersections is plotted at the top with black bars. *PRR/NLR* genes activated in *mom1* (Cambiagno et al., 2018) are highlighted as blue fractions of the bars. References indicate the transcriptomes analyzed. **(B)** Box-plot graphs of the RPKM (reads per kilobase of exon per million reads mapped) for the immune receptors genes *ADR1* and *RLK7* from datasets used in **(A)**. Asterisks indicate significant differences (FDR < 0.05, FC > 1; DEseq2 analysis). **(C)** *RLK7* and *ADR1* transcript levels quantified by RT-qPCR in WT, *mom1-1* and *miniMOM1* leaves 1 day after root-applied 1mM AZA, 300 µM BABA, 1mM PIP, or mock solutions. Values represent average +/- standard error of three independent experiments (each data point with 3 leaves from 3 different plants pooled together for RNA extraction). *ELF1α* was used as a reference gene. Expression levels are relative to mock-treated WT plants. Individual data points are presented as scatter-dots. Different letters indicate significant differences among samples (P < 0.05, ANOVA, Fisher’s LSD test).

The SAR signals AZA and PIP down-regulate the *MOM1* transcripts systemically (**Figure 4**), and MOM1 deficiency triggers the activation of several *NLR*/*PRR* genes (*MOM1-NLR/PRRs*) (Cambiagno et al., 2018). Therefore, *MOM1-NLR/PRRs* may become upregulated during SAR or priming induction. To explore this idea, we made use of publicly available transcriptome data to search for shared induced immune receptor genes among *mom1* mutants and SAR- or PIP/NHP-primed systemic tissues (Bernsdorff et al., 2016; Cambiagno et al., 2018; Hartmann et al., 2018; Baum et al., 2019; Yildiz et al., 2021). We first analyzed the expression of all *NLR/PRRs* in each transcriptome and selected the upregulated genes (FDR < 0.05, FC > 1; see section “Materials and Methods”). As expected, many induced receptors genes are shared between SAR- or PIP/NHP-primed datasets (**Figure 5A**). Next, we detected the common *NLR/PRRs* genes between the different transcriptomes and those reported as *MOM1-NLR/PRRs* (Cambiagno et al., 2018). Interestingly, more than 50% of the *MOM1-NLR/PRRs* (13 out of 25) are also induced in at least 1 transcriptome dataset analyzed (**Figure 5A**, blue fractions of the bars). Moreover, 40% of these *NLR/PRRs* are upregulated in 4 out of 6 analyzed transcriptomes and several of them encode for receptors characterized at the biological level. This suggests that MOM1 may control a subset of *NLR/PRRs* genes during the activation of systemic resistance or defense priming.

Two of these shared *MOM1-NLR/PRRs* genes, *RLK7* and *ADR1*, that also recover their repression in *miniMOM1* plants (Cambiagno et al., 2018), might be playing an important role(s) in plant resistance (Bonardi et al., 2011; Hou et al., 2014; Jubic et al., 2019). Thus, we individually analyzed their induction in the SAR- or PIP/NHP-transcriptomes. As observed in **Figure 5B**, both genes showed enhanced expression in all tested primed systemic tissues, with *RLK7* showing a significant induction in most datasets. Considering this and the potential key role on defenses amplification of *RLK7* and *ADR1*, we experimentally determined their expression in leaves of wild-type, *mom1-1* and *miniMOM1* plants one day after root-treatment with AZA, BABA and PIP as previously described (**Figure 4B**). We found that in WT plants all the inducers enhanced *RLK7* and *ADR1* expression (**Figure 5C**), supporting the data obtained from transcriptome analysis (**Figure 5B**). In the *mom1* mutant we noticed a trend towards induction of *RLK7* and *ADR1* in mock treated samples, consistently with previous results (Cambiagno et al., 2018). None of the three inducers significantly alters the expression of these genes in this mutant. Unexpectedly, *miniMOM1* plants exhibited a marked reduction in the levels of *RLK7* and *ADR1* transcripts after mock, AZA, PIP or BABA treatments compared to both WT and *mom1* plants.

Together, these results strongly support MOM1 as a factor implicated in SAR, probably facilitating the upregulation of several *NLR/PRRs* as a form of defense priming against pathogens.

## Discussion

The immunological memory or priming is a fundamental process for plants to resist disease (Martinez-Medina et al., 2016). However, there is scarce evidence of the molecular basis and factors underlying its induction. Here, we provide data that strongly support the chromatin factor and TGS-regulator MOM1 as an important component of the priming against *Pseudomonas* sp. pathogen in *Arabidopsis*. *mom1* mutants showed an increased primary root susceptibility to the inhibitory growth effect of the priming inducers AZA, BABA and PIP under optimal *in vitro* conditions (**Figure 2**). Contrary, plants expressing a gain-of-function version of MOM1 (*miniMOM1*) showed a reduced response. Moreover, *mom1* mutant showed a basal primed-like phenotype while *miniMOM1* plants were impaired in AZA-, PIP- or BABA-mediated systemic resistance against *Pma*DG3 (**Figure 3**). Importantly, the treatment with all these priming inducers reduced the *MOM1* expression in wild-type plants, while the *miniMOM1* transgene transcript levels did not change (or even increased) in *miniMOM1* plants (**Figure 4**). Additionally, we found that many of the immune receptors known to be induced in *mom1* mutants [*MOM1-NLR/PRRs*; (Cambiagno et al., 2018)], were also upregulated in PIP- and SAR-primed plants (**Figure 5**). Furthermore, in adult *miniMOM1* plants the *MOM1-NLR/PRRs* genes *RLK7 and ADR1* showed lower basal levels compared to the WT. Altogether our results position MOM1 as a negative regulator of the primed state induced by AZA, BABA and PIP in *Arabidopsis*. Considering these and our previous results (Cambiagno et al., 2018), we propose that under priming-inducing conditions MOM1 levels are reduced as a form of sensitization to biotic stresses. In plants exposed to non-sterile or stressful environmental conditions, the decrease in MOM1 facilitates the upregulation of important immune receptors (e.g., RLK7 and ADR1), improving the perception of future attacking pathogens and/or the amplification of the plant defense responses.

### *mom1*-activated defenses are associated to the environmental growth conditions

We have previously suggested that the basal activation of defenses observed in *mom1* could be determined by aging or by the higher susceptibility of the mutant to some stresses occurring during development (Cambiagno et al., 2018). However, using optimal sterile conditions of gas exchange and minimal stress (Xu et al., 2019), we were unable to detect activation of *RLK7*, *ADR1*, *PR1* and *ICS1* genes during *mom1-1* plants grown, until the transition to flowering (**Figure 1**). Therefore, these results indicate that some undefined abiotic and/or biotic stress triggers defense induction in *mom1* independently of its developmental condition. Interestingly, in wild-type plants an increase of the disease resistance is related to aging, the so-called age-related resistance (ARR) (Kus et al., 2002). ARR is neither associated with flowering transition nor with plant senescence, and the components implicated are largely unknown (Carella et al., 2015; Wilson et al., 2017). Considering this, it is possible that ARR is related to a reduction of MOM1 and, thus, a continuous/gradual increase of the plant responsiveness (and resistance) to environmental stresses. This is supported by the fact that under ideal growth conditions, the wild-type plants did not show an increase in defense marker genes either (**Figure 1**). Future analysis of the MOM1 level in mutants and/or cultivars/ecotypes with enhanced ARR (or basal resistance) growing in “normal” or germ-free and controlled environments, will shed light into this possibility.

### MOM1 regulates root responses to AZA, BABA and PIP

AZA-, BABA- and PIP-mediated defense priming correlates with root growth inhibition [this work, (Wu et al., 2010; Bouain et al., 2018; Cecchini et al., 2019; Janotík et al., 2022)]. Each inducer requires different defense signaling components (Wu et al., 2010; Mauch-Mani et al., 2017; Bouain et al., 2018; Cecchini et al., 2019; Hartmann and Zeier, 2019; Vlot et al., 2021). However, MOM1 could be common to all these pathways since *mom1* roots growth showed hyper-susceptibility to all the inducers, while the expression of the *miniMOM1* gain-of-function version made plants less responsive to them (**Figure 2**). Moreover, it was shown that AZA signaling factors are needed for the root-induced ISR (Cecchini et al., 2015b). Thus, MOM1 might also play a role in the known relationship between principal root inhibition and the systemic resistance promoted by soil-beneficial microbes (Contreras-Cornejo et al., 2009; Ortiz-Castro et al., 2011; Zamioudis et al., 2013; Spaepen et al., 2014). An attractive idea is that MOM1 is involved in the fine-tuning of the known growth-defense tradeoff (Huot et al., 2014). In support, one of the *MOM1*-*NLR/PRRs*, RLK7, was recently identified as perceiving Pip1 and TOLS2/PIPL3 peptides, known to amplify immunity and influence the morphology of the roots, respectively (Hou et al., 2014; Toyokura et al., 2019). Moreover, the auxin-signaling, proposed to balance growth and immunity, affects the trafficking of RLK7 receptor (Stringlis et al., 2018; Toyokura et al., 2019). The analysis of MOM1 and the priming inducers level in different biotic interactions will provide answers about the possible role of MOM1 in balancing growth and defense.

### MOM1 is an epigenetic factor implicated in the immunological memory

It is believed that for the establishment of the primed state epigenetic factors are playing key roles by regulating the chromatin structure and marks (Mozgová et al., 2015; Crisp et al., 2016; Lämke and Bäurle, 2017; Alonso et al., 2019; Wilkinson et al., 2019; Hannan Parker et al., 2022). In fact, many epigenetic factors appear to function as negative regulators of priming. However, mutants lacking these components use to show constitutively activated defenses, reduced growth and/or pleiotropic phenotypes (López et al., 2011; Luna et al., 2012; Singh et al., 2014; Liu et al., 2018; Cambiagno et al., 2021; Noh et al., 2021). None of these traits have been detected in *mom1* plants. Moreover, we provide strong evidence involving MOM1 in the negative regulation of the defense priming induced by AZA, BABA and PIP. The primed state, like all other phenotypes described for *mom1*, depends on the CMM2 domain (this work; Cambiagno et al., 2018), suggesting an association between defense priming and the MOM1-mediated pericentromeric heterochromatin silencing capacity. In agreement, *PRR/NLRs* and defense genes have not been described as direct targets of MOM1, nor included as MOM1 targets containing bivalent epigenetic marks and chromatin states intermediate between hetero- and euchromatin (Habu et al., 2006; Numa et al., 2010; Yokthongwattana et al., 2010). A mechanism involving *trans* regulation of p*TE* and distal *PRR/NLR* genes by common sRNAs was suggested to operate in wild-type plants infected with *Pseudomonas* sp., where *TSI* is transiently expressed and then re-silenced through RdDM (Cambiagno et al., 2018). Although the activation of p*TE* has a different origin in *mom1* than in wild-type plants, p*TE* and distal *PRR/NLRs* may also be co-regulated in this plant, since RdDM-dependent p*TE* silencing by CMM2 expression abolishes defense induction in *miniMOM1* plants. However, we here confirm that the activation of p*TE* is not sufficient to trigger defense gene induction in *mom1* (**Figure 1**). Thus, differences in the component levels, assembly, maintenance, or targeting of multi-protein p*TE* silencing complexes containing MOM1 (CMM2), could explain the priming phenotypes observed in wild-type and *miniMOM1* plants. In fact, MOM1 acts together with the SUMO E3 ligase-like proteins PIAL1 and PIAL2 to maintain transcriptional silencing of heterochromatic p*TE*s and interacts with these proteins through its CMM2 domain, forming a high molecular mass complex *in vivo* (Han et al., 2016). How this complex mediates *TE* silencing is unknown. Although MOM1 is a target for sumoylation, this modification is not required for p*TE* silencing. Still, sumoylation of other components of the silencing machinery may occur (Han et al., 2016). Another possible component of the MOM1 multi-protein p*TE* silencing complex(es) is the chromatin remodeler DDM1. It was shown that MOM1 functions with DDM1 in resetting abiotic stress memory (Iwasaki and Paszkowski, 2014; Iwasaki, 2015) and, interestingly, DDM1 also maintains silencing of pericentromeric heterochromatin (Habu et al., 2006; Iwasaki and Paszkowski, 2014; Furci et al., 2019). Moreover, it was recently shown that the hypomethylation at pericentromeric regions in *ddm1* mutants correlates with disease resistance associated with the priming of unlinked defense genes (Furci et al., 2019; Wilkinson et al., 2019). Furthermore, *DDM1* transcription levels are reduced during pathogen infections [unpublished results from (Furci et al., 2019); Genevestigator and eFP Browser (Winter et al., 2007; Hruz et al., 2008)]. Thus, one possibility is that MOM1 and DDM1 act together during the priming and systemic resistant programs as important negative regulators of immunological memory. Future analysis using optimal germ-free growth conditions and/or gain-of-function plants, might help explain the role of PIAL1/2, DDM1, and other epigenetic factors in plants defense priming.

### MOM1 act as a priming factor by regulating immune receptors level

The reduction of MOM1 in response to AZA, BABA and PIP, may trigger the induction of *MOM1*-*NLR/PRRs* in a non-sterile environment, enhancing the perception of infections as a form of priming. This is supported by the fact that *miniMOM1* plants, that complement *mom1* p*TEs* overexpression and reduce the activation of *MOM1*-*NLR/PRR* in soil-grown adult plants [**Figures 1**, **5**; (Čaikovski et al., 2008; Cambiagno et al., 2018)], were unresponsive (or less responsive) to all the priming inducers (**Figures 2**, **3**). Since AZA and PIP are known to be involved in SAR and/or ISR (Cecchini et al., 2019; Vlot et al., 2021), it is also possible that MOM1 acts as a regulator of the memory associated to these systemic resistance programs. If this is true, it is expected that the MOM1-*NLR*/*PRR* genes will be upregulated during these defense programs. In agreement, we found that half of the reported MOM1-*PRR/NLRs* were also induced in SAR- and/or PIP-primed systemic tissues transcriptomes (**Figure 5**) (Bernsdorff et al., 2016; Cambiagno et al., 2018; Hartmann et al., 2018; Baum et al., 2019; Yildiz et al., 2021). It is also anticipated that all or some of the MOM1-*NLR*/*PRR* will be important in making the plant defense responses faster/stronger and/or more sustained after a second infection. In fact, two of these immune receptor genes, *RLK7* and *ADR1*, might play key roles on the primed state. RLK7 amplifies the defense responses associated to the PTI (Hou et al., 2014). While ADR1 is a *helper* NLR that perceives the activity of other NLRs, playing a main role in ETI (Bonardi et al., 2011; Baggs et al., 2017; Jubic et al., 2019). Thus, an increase of these immune receptor levels constitutes an ideal manner to boost pathogens recognition. Future analysis of the primed state in *mom1 rlk7*/*adr1* (or other *MOM1*-*NLR*/*PRRs*) double mutants may show the importance of these receptors for the MOM1-mediated immunological memory.

In summary, we have shown that the chromatin and TGS regulator MOM1 plays an important role in the primed state induced by AZA, PIP and BABA. Because these priming inducers are naturally produced in plants and AZA and PIP (or NHP) are proposed mobile defense signals, MOM1 might be a general component of the immune memory associated to different systemic resistance programs. Since MOM1 is highly conserved between plant species, and *mom1* mutant plants do not show visible growth defects, our work could also help in developing crops with an improved yield when growing under biotic stress conditions.

## Data availability statement

The datasets presented in this study can be found in online repositories. The names of the repository/repositories and accession number(s) can be found in the article/**Supplementary Material**.

## Author contributions

NC and MA conceived and designed the experiments. JM, MP, IL, DC, and NC performed the experiments. JM, MP, IL, DC, MA, and NC analyzed and interpreted data. NC and MA wrote the paper. All authors contributed to the article and approved the submitted version.
