# Supplementary material for: The Arabidopsis chromatin regulator MOM1 is a negative component of the defense priming induced by AZA, BABA and PIP
Source: Front Plant Sci. 2023 May 9;14:1133327. doi: 10.3389/fpls.2023.1133327 (PMC10203520; doi:10.3389/fpls.2023.1133327)
Supplement: Supplementary file 1 [file Table_1.docx]

**Supplementary table 1.** Primer list used in this study

| **Primers** | | | |
| --- | --- | --- | --- |
| Name | | Sequence (5’-3’) | Purpose |
| PR1_Fw | AATACTGGCTATTCTC | | qPCR for *PR1* |
| PR1_Rv | AGGGAAGAACAAGAGCAACTA | | qPCR for *PR1* |
| ICS1_Fw | TGGTTAGCGTTGCTGGTATC | | qPCR for *ICS1* |
| ICS1_ Rv | CATTCAACAGCGATCTTGCC | | qPCR for *ICS1* |
| ADR1_Fw | AGTTTGTGAGCTGCCATGTCTA | | qPCR for *ADR1* |
| ADR1_Rv | CTCAAGACTCCCTAGCTTTCCA | | qPCR for *ADR1* |
| RLK7_Fw | ACCGATAGAGGCAGAGTTTGG | | qPCR for *RLK7* |
| RLK7_Rv | TGTCCACAATCTCCATCACACT | | qPCR for *RLK7* |
| elF1a-F FW | AGGTCCACCAACCTTGACTG | | qPCR for *EF1α* |
| elF1a-F RV | GAGACTCGTGGTGCATCTCA | | qPCR for *EF1α* |
| CMM2_Fw | CCAAGTAGAGCGGTCAAC | | MOM1; qPCR for CMM2 domain |
| CMM2_Rv | TTACCCCTTGGAGCTCCT | | MOM1; qPCR for CMM2 domain |
| TSI_Fw | TGGTTCACCAGATAAGCTCAGTGCCCTC | | qPCR for *TSI* |
| TSI_Rv | GGGAATGGTATCAGATCCTAACAGTGTGG | | qPCR for *TSI* |
|  |  | |  |

FW/RV: forward and reverse primers.
